# Whole transcriptome sequencing for revealing the pathogenesis of sporotrichosis caused by *Sporothrix globosa*

**DOI:** 10.1038/s41598-023-50728-7

**Published:** 2024-01-03

**Authors:** Zhe Liu, Su-Shan Li, Gui-Yun Zhang, Sha Lv, Shuang Wang, Fu-Qiu Li

**Affiliations:** Department of Dermatology, The Second Hospital of Jilin University, No. 218 Ziqiang Road, Nanguan District, Changchun, 130041 Jilin Province China

**Keywords:** Computational biology and bioinformatics, Microbiology

## Abstract

This study aimed to investigate the molecular mechanism of sporotrichosis and identify possible novel therapeutic targets. Total RNA was extracted from skin lesion samples from sporotrichosis patients and used to construct a long-chain RNA transcriptome library and miRNA transcriptome library for whole transcriptome sequencing. The differentially expressed genes (DEGs) between the groups were identified, and then Gene Ontology, Kyoto Encyclopedia of Genes and Genomes, and Gene Set Enrichment Analysis enrichment analyses were performed based on the DEGs. An lncRNA-miRNA-mRNA ceRNA network was constructed. The expressions of JAK/STAT pathway-related proteins were detected in the patient and control tissues using RT-qPCR and Western blot analysis. Enrichment analysis showed that the DEGs were mainly enriched in various infectious diseases and immune response-related signaling pathways. Competing endogenous RNA network analysis was performed and identified the hub lncRNAs, miRNAs, and mRNAs. Compared with the control group, the mRNA expressions of SOCS3, IL-6, and JAK3 were significantly upregulated, while the expression of STAT3 did not change significantly. Also, the protein expressions of SOCS3, IL-6, JAK3, and STAT3, as well as phosphorylated JAK3 and STAT3, were significantly upregulated. We identified 671 lncRNA DEGs, 3281 mRNA DEGs, and 214 miRNA DEGs to be involved in *Sporothrix globosa* infection. The study findings suggest that the JAK/STAT pathway may be a therapeutic target for sporotrichosis.

## Introduction

Sporotrichosis, a subcutaneous fungal infectious disease that is widely distributed worldwide, is an infection caused by a dimorphic fungus called *Sporothrix*. Infection may occur in the skin, subcutaneous tissue, mucous membrane, and nearby lymphoid tissues, both in humans and animals^1–3^.

Sporotrichosis is prevalent in China, especially in the northeast region, mainly caused by *Sporothrix globosa* infection^4–7^. Sporotrichosis occurs both in males and females regardless of age and it can be divided into four types according to the clinical manifestations: lymphocutaneous, fixed cutaneous, disseminated cutaneous, and extracutaneous^8^, with the former two the most common^9^, while the latter two may be relatively less common but more serious^10–15^.

Although sporotrichosis has been known about for over 100 years, there is a limited variety of clinical treatments available. The current main treatments include the use of immunomodulators, such as potassium iodide, antifungal drugs, and physical treatments, such as heat therapy, cold therapy, and photodynamic therapy^16–22^, but more effective therapeutic approaches with little adverse reactions are needed and may be expected in the future. Generally, the duration of sporotrichosis treatment is about 3–6 months, although sometimes, an additional 4–6 weeks of maintenance treatment are needed to ensure mycological cure after a clinical complete remission. However, such long-term medication can lead to a great economic and psychological burden on sporotrichosis patients. Moreover, high mortality has been reported in disseminated sporotrichosis patients^23^. Despite its global distribution, the pathogenic mechanism of sporotrichosis remains unclear, and more research is needed to explore the pathogenesis and underlying molecular mechanism of the disease to aid finding new therapeutic targets.

High-throughput screening approaches, such as transcriptomics, proteomics, and metabolomics, can help clarify the development of biological events and help identify the key biomolecules involved in biological processes. These high-throughput approaches can facilitate the identification of biomarkers, contribute to an understanding of the dynamics of an infection and the host immune responses, and help highlight the key pathways involved in the infection process^24^. Transcriptomics is the analysis of all RNA transcripts, including both coding and noncoding RNAs (ncRNAs), in cells or tissues. Studies have shown that ncRNAs are involved in cell proliferation and differentiation, epigenetic modification, apoptosis, and the complex pathogenesis of various diseases^25^. With the development of high-throughput sequencing technology, transcriptomics is increasingly being used to study the pathogenic mechanisms of emerging diseases and some diseases whose pathogenesis remains unclear^24,26^. During the early outbreak of COVID-19, for instance, there was scarce understanding of the virus, and so medical teams tried a large number of methods to deal with COVID-19 infection, mostly with unsatisfactory results, until scientists worldwide carried out transcriptomic studies to explore and analyze the possible genes involved in the infection, with the aim to identify a novel therapeutic target for COVID-19^27^.

In the present study, we aimed to investigate the molecular mechanism of sporotrichosis and identify novel possible therapeutic targets through transcriptomic analysis of the skin lesions of sporotrichosis patients.

## Materials and methods

### Patients

Samples from the skin lesions of 15 patients diagnosed with sporotrichosis in our hospital from December 2021 to December 2022 were collected. The diagnosis of sporotrichosis was confirmed based on histopathological examination and mycological culture (of the purulent secretion/tissue mass at the lesion site). All the patients were found to be infected with *Sporothrix globosa*, identified by mycelial-to-yeast phase-conversion culture and molecular identification methods^28^. The inclusion criteria for the intervention group were patients aged ≥ 18 years old and diagnosed with fixed sporotrichosis or (unilateral) lymphatic sporotrichosis. The exclusion criteria were patients with multifocal or disseminated or extracutaneous sporotrichosis, sporotrichosis accompanied by other infectious diseases, kidney or liver disease, or who had received steroid or antifungal treatment 2–8 weeks before sample collection, had received immunosuppressive treatment 4 weeks before sample collection, or were suffering from systemic diseases, such as cardiovascular disease, bacterial pneumonia, rheumatism, diabetes, gout, or a tumor. Normal skin tissue samples were collected from nine patients who had undergone blepharoplasty in the same hospital. The exclusion criteria for the control subjects were individuals with a history of chronic skin disease, tumor, or other systemic diseases. The Ethics Committee of The Second Hospital of Jilin University approved the study and waived the requirement for written informed consent due to the retrospective nature of the study.

### RNA extraction, library preparation, and sequencing

Total RNA was extracted from the collected tissue samples using TRIzol (Thermo Fisher, Waltham, MA, USA) according to the manufacturer’s instructions. Ribosomal RNA was removed using the MGIEasy rRNA depletion kit (MGI, China). The RNA integrity numbers (RINs) of the RNA samples ranged from 6.6 to 8.5. The first cDNA was synthesized using the MGIEasy RNA Directional Library Prep Set (MGI, China). The RNA library preparations and sequencing reactions were conducted at MGI (Wuhan, China). For miRNA construction, total RNA was separated by polyacrylamide gel electrophoresis (PAGE) and RNA 18–30 nt in length was purified. The miRNA library preparations and sequencing reactions were conducted at MGI (Wuhan, China).

### Quality control, mapping, and transcriptome assembly

Clean reads were obtained after filtering the raw data and then the Q20 (a sequencing error rate of 1 in 100 with an accuracy of 99%) and Q30 (a sequencing error rate of 1 in 1000 with an accuracy of 99.9%) rates, and GC contents of the clean data were calculated to evaluate the quality of the clean data. All the subsequent analyses were thus based on high-quality clean data. Then the clean data were mapped to the reference genome (Homo_sapiens GCF_000001405.39_GRCh38.p13) using Bowtie2^29^. The matched reads were calculated as fragments per kilobase of transcript per million mapped reads (FPKM) for further analysis using RSEM software^30^. The raw sequence data have been deposited in the Genome Sequence Archive in National Genomics Data Center, China National Center for Bioinformation / Beijing Institute of Genomics, Chinese Academy of Sciences (GSA-Human: HRA003986) and are publicly accessible at https://ngdc.cncb.ac.cn/gsa-human.

### Analysis of the differentially expressed genes

Principal component analysis (PCA) and heatmap plots were utilized to obtain an overview of the expression profiles of the long noncoding, messenger, and micro RNA (lncRNA, mRNA, and miRNA). The DEseq2 method was used for differential gene expression analysis^31^. The differentially expressed genes (DEGs) were defined as genes having an adjusted p (Q) value < 0.05 and an absolute value of log 2 (fold change) > 1 and presented using volcano plots.

### Enrichment analysis

Gene Ontology (GO) enrichment analysis of the DEGs was performed to explore the potential roles of the differentially expressed mRNAs. The *P*-value was adjusted by the Benjamini and Hochberg correction, and GO terms with a false discovery rate (FDR) < 0.05 were considered to be significantly enriched. Kyoto Encyclopedia of Genes and Genomes (KEGG) function enrichment analysis of the DEGs was performed to identify the associated biochemical and signal transduction pathways^32^. Gene Set Enrichment Analysis (GSEA) was performed using Dr. Tom (a data analysis platform from BGI) with the official software package of GSEA.

### Competing endogenous RNA (ceRNA) network construction

The target miRNAs of the differential lncRNAs were predicted using the miRcode and lncBase databases. Then, the miRNAs intersecting with the differential miRNAs were obtained and the target mRNAs of the intersected miRNAs were predicted using the RNAHybrid, miRTarBase, and TargetScan databases, and the mRNAs intersecting with the differential mRNAs were thus obtained. Finally, a ceRNA network was constructed using Cytoscape software through the obtained differential lncRNA-intersection and miRNA-intersection mRNAs.

### Quantitative reverse transcription polymerase chain reaction (qRT-PCR)

Total RNA was extracted from the collected tissues using TRIzol (Thermo Fisher, Waltham, MA, USA) according to the manufacturer’s instructions. cDNA was synthesized via reverse transcription of the total RNA using the PrimeScript™ RT Reagent Kit (TAKARA, Tokyo, Japan). qPCR was performed on the real-time PCR system ViiA7 (ABI, USA) using HieffTM qPCR SYBR Green Master Mix (No Rox) (Yeasen, Shanghai, China) with the following procedure: initiation at 95 °C for 5 min, followed by 40 cycles of 95 °C for 10 s, and 60 °C for 30 s. The primers are presented in Supplementary Table 1. The relative expression of mRNAs was determined using the 2^−ΔΔCt^ method and was normalized to GAPDH.

### Western blot analysis

Tissues were lysed in RIPA buffer (Servicebio, Wuhan, China) containing a protease inhibitor cocktail (Servicebio). Protein samples were separated on 12% sodium dodecyl sulfate–polyacrylamide gels by electrophoresis and transferred onto nitrocellulose membranes (Servicebio). The membranes were blocked and incubated overnight at 4 °C with primary antibodies against IL-6, JAK3, p-JAK3, STAT3, p-STAT3, SOCS3 (dilution 1:1000 for all), and β-actin (dilution 1:2000). The membranes were then incubated with horseradish peroxidase (HRP)-conjugated secondary antibodies (Servicebio; 1:5000) for 1 h at room temperature. The protein bands were visualized using an enhanced chemiluminescence (ECL) kit (Servicebio), imaged using a CLINX 6100 imaging system (ClINX, Shanghai, China), and quantified with AIWBwell™ software (Servicebio). β-actin was used as an internal control.

### Statistical analysis

Data are presented as the mean ± standard deviation, and comparisons between the two groups were performed using *t* tests. The statistical analysis was performed using GraphPad Prism 8.0 software. *P* < 0.05 was considered statistically significant.

### Informed consent

Patient consent was waived by the Ethics Committee of The Second Hospital of Jilin University due to the study being a retrospective study and as the identity of all the patients remained undisclosed.

## Institutional Review Board Statement

 The Ethics Committee of The Second Hospital of Jilin University approved the study [approval number: 2021(188)] and waived the requirement for written informed consent. All methods were performed in accordance with the Declaration of Helsinki.

## Results

### Demographic characteristics of the patients with sporotrichosis and the healthy controls

Samples were collected from 15 patients with sporotrichosis and 9 healthy controls. Five samples from the patients with sporotrichosis and three control samples were used for the RNA sequencing. The demographic characteristics of all the study participants are shown in Supplementary Table 2.

### Expression patterns of the RNAs as assessed by PCA and heatmap plots

According to the PCA analysis, the patients with sporotrichosis could all be clearly distinguished from the healthy controls by their lncRNA (Supplementary Fig. 1A), mRNA (Supplementary Fig. 1B), and miRNA (Supplementary Fig. 1C) expression profiles. Moreover, the heatmap plots for the lncRNA (Supplementary Fig. 1D), mRNA (Supplementary Fig. 1E), and miRNA (Supplementary Fig. 1F) expression profiles also showed distinct expression signatures.

### Differential expression of LncRNAs, mRNAs, and miRNA in patients with sporotrichosis

**Figure 1:**
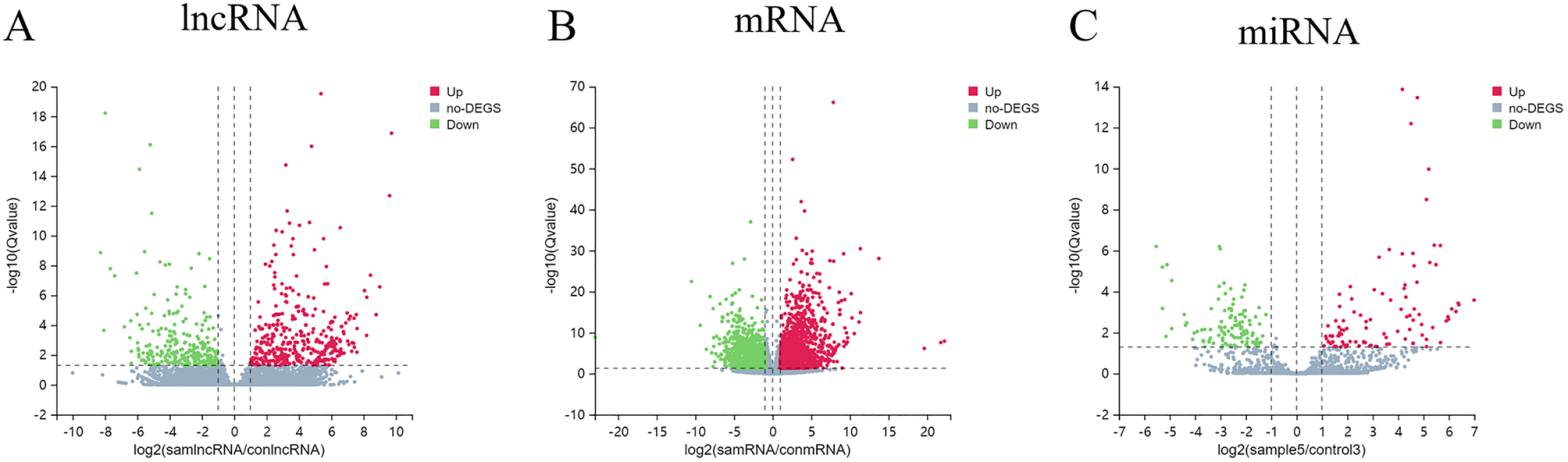
Volcano plots of all the RNA transcripts showing the differentially expressed genes (DEGs). (**A**) lncRNA; (**B**) mRNA; (**C**) miRNA.

Volcano plots were used to show the differentially expressed lncRNAs, mRNAs, and miRNAs in patients with sporotrichosis compared to the healthy controls (Fig. 1). In total, 51 706 lncRNAs were tested, among which 671 lncRNAs were differentially expressed, with 392 upregulated and 279 downregulated. The top 10 upregulated and downregulated lncRNAs are listed in Supplementary Table 3. Also, a total of 18,561 mRNAs were tested, among which 3281 mRNAs were differentially expressed, with 1693 upregulated and 1588 downregulated. The top 10 upregulated and downregulated mRNAs are listed in Supplementary Table 4. In total, 1933 miRNAs were tested, among which 214 miRNAs were differentially expressed, with 99 upregulated and 115 downregulated. The top 10 upregulated and downregulated miRNAs are listed in Supplementary Table 5.

### GO functional enrichment of the differentially expressed mRNAs

**Figure 2:**
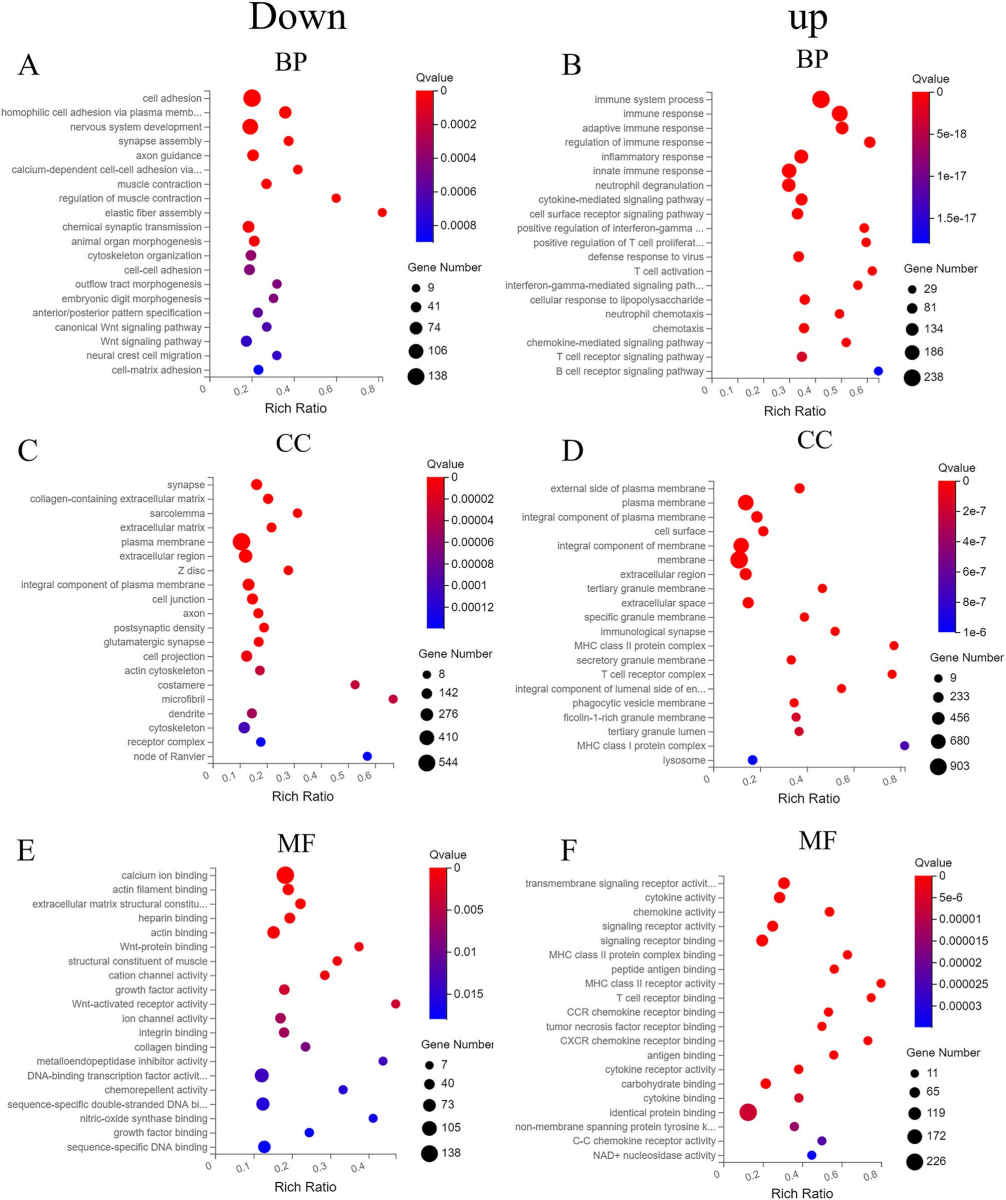
GO enrichment analysis of the downregulated (**A**,**C**,**E**) and upregulated (**B**,**D**,**F**) mRNA DEGs. (**A**,**B**) Biological processes (BPs); (**C**,**D**) cell components (CCs); (**E**,**F**) molecular functions (MFs).

For the GO enrichment analysis, the genes were classified into three categories: biological processes (BPs), molecular functions (MFs), and cellular components (CCs). Among the BPs, the downregulated differentially expressed mRNAs were responsible for cell matrix adhesion, neural crest cell migration, and the Wnt signaling pathway (Fig. 2A), while the upregulated differentially expressed mRNAs were responsible for the B-cell receptor signaling pathway, T-cell receptor signaling pathway, and chemokine-mediated signaling pathway (Fig. 2B). Among the CCs, the downregulated differentially expressed mRNAs were responsible for the node of Ranvier, receptor complex, and cytoskeleton (Fig. 2C), while the upregulated differentially expressed mRNAs were responsible for the lysosomes, MHC class I protein complexes, and tertiary granule lumen (Fig. 2D). Among the MFs, the downregulated differentially expressed mRNAs were responsible for sequence-specific DNA binding, growth factor binding, and nitric-oxide synthase binding (Fig. 2E), while the upregulated differentially expressed mRNAs were responsible for the NAD + nucleosidase activity, C–C chemokine receptor activity, and non-membrane spanning protein tyrosine kinase activity (Fig. 2F).

### KEGG pathway enrichment of the differentially expressed mRNAs

**Figure 3:**
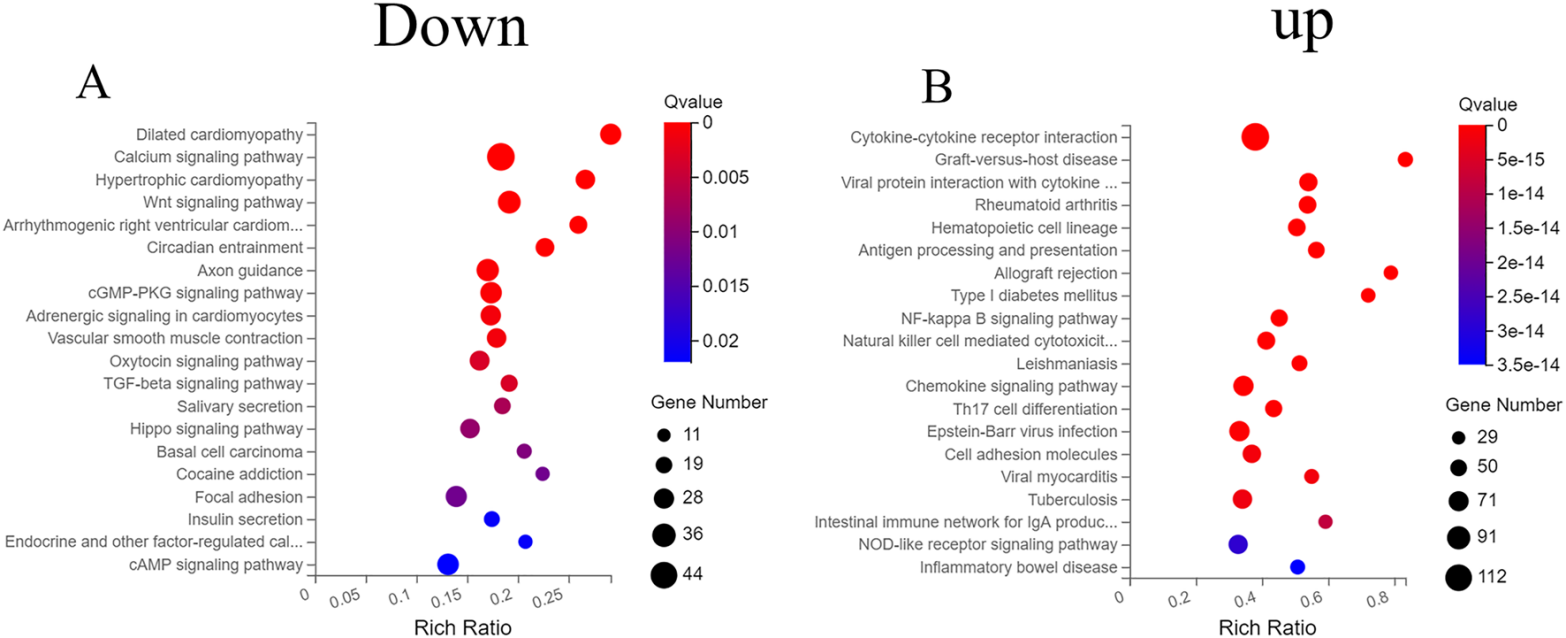
KEGG enrichment analysis of the downregulated (**A**) and upregulated (**B**) mRNA DEGs^32^.

KEGG enrichment analysis showed that the downregulated differentially expressed mRNAs were mainly enriched in the cAMP signaling pathway, Hiippo signaling pathway, TGF-beta signaling pathway, and Wnt signaling pathway, while the upregulated mRNA were mainly involved in the nucleotide oligomerization domain (NOD)-like receptor signaling pathway, natural killer (NK) cell-mediated cytotoxicity, antigen processing and presentation, and nuclear factor (NF)-kappa B signaling pathway (Fig. 3).

### GESA enrichment analysis

**Figure 4:**
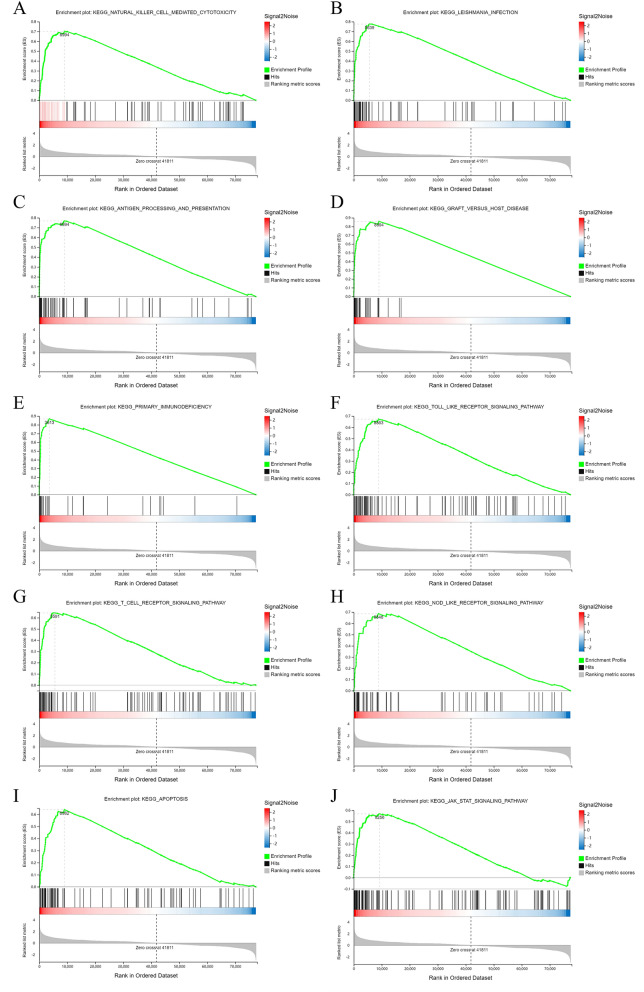
GESA enrichment analysis of all the mRNA DEGs. (**A**) Natural killer-cell-mediated cytotoxicity; (**B**) Leishmania infection; (**C**) antigen processing and presentation; (**D**) graft versus host disease; (**E**) primary immunodeficiency; (**F**) toll-like receptor signaling pathway; (**G**) T-cell receptor signaling pathway; (**H**) NOD-like receptor signaling pathway; (**I**) apoptosis; (**J**) JAK/STAT signaling pathway.

Next, we performed GESA enrichment analysis on the differentially expressed mRNAs. As shown in Fig. 4, the differentially expressed mRNAs were mainly enriched in NK cell-mediated cytotoxicity, antigen processing and presentation, primary immunodeficiency, the toll-like receptor (TLR) signaling pathway, T-cell receptor (TCR) signaling pathway, NOD-like receptor signaling pathway, apoptosis, and Janus kinase/signal transducer and activator of transcription (JAK/STAT) signaling pathway.

### Construction of a ceRNA interaction network identifying the hub lncRNAs, miRNAs, and mRNAs

An lncRNA-miRNA-mRNA co-expression network was constructed for the differentially expressed lncRNAs, miRNAs, and mRNAs. In this ceRNA interaction network, the key lncRNAs included LOC105374338, LOC105370792, and URS0001BCE66F, while the key miRNAs included miR-143-5p, miR-20b-5p, miR-31-5p, and miR-1268a, and the key mRNAs included JAK3, SLC4A1, ESR1, and GMAT (Supplementary Fig. 2).

### Validation of the four DEGs DKK1, ACTG2, JAK3, and SLC7A11

To further validate the expression changes in sporotrichosis patients, qRT-PCR was performed to analyze the expression of four genes (DKK1, ACTG2, JAK3, and SLC7A11) in an additional eight skin lesions samples of sporotrichosis patients and four control skin samples. The results showed that compared with the control group, the expressions of DKK1 and ACTG2 were significantly downregulated while the expressions of JAK3 and SLC7A11 were significantly upregulated in the tissues of the sporotrichosis patients (Supplementary Fig. 3), consistent with the sequencing data.

### Expression changes of the JAK/STAT signaling pathway-related proteins (IL-6, JAK3, STAT3, and SOCS3)

**Figure 5:**
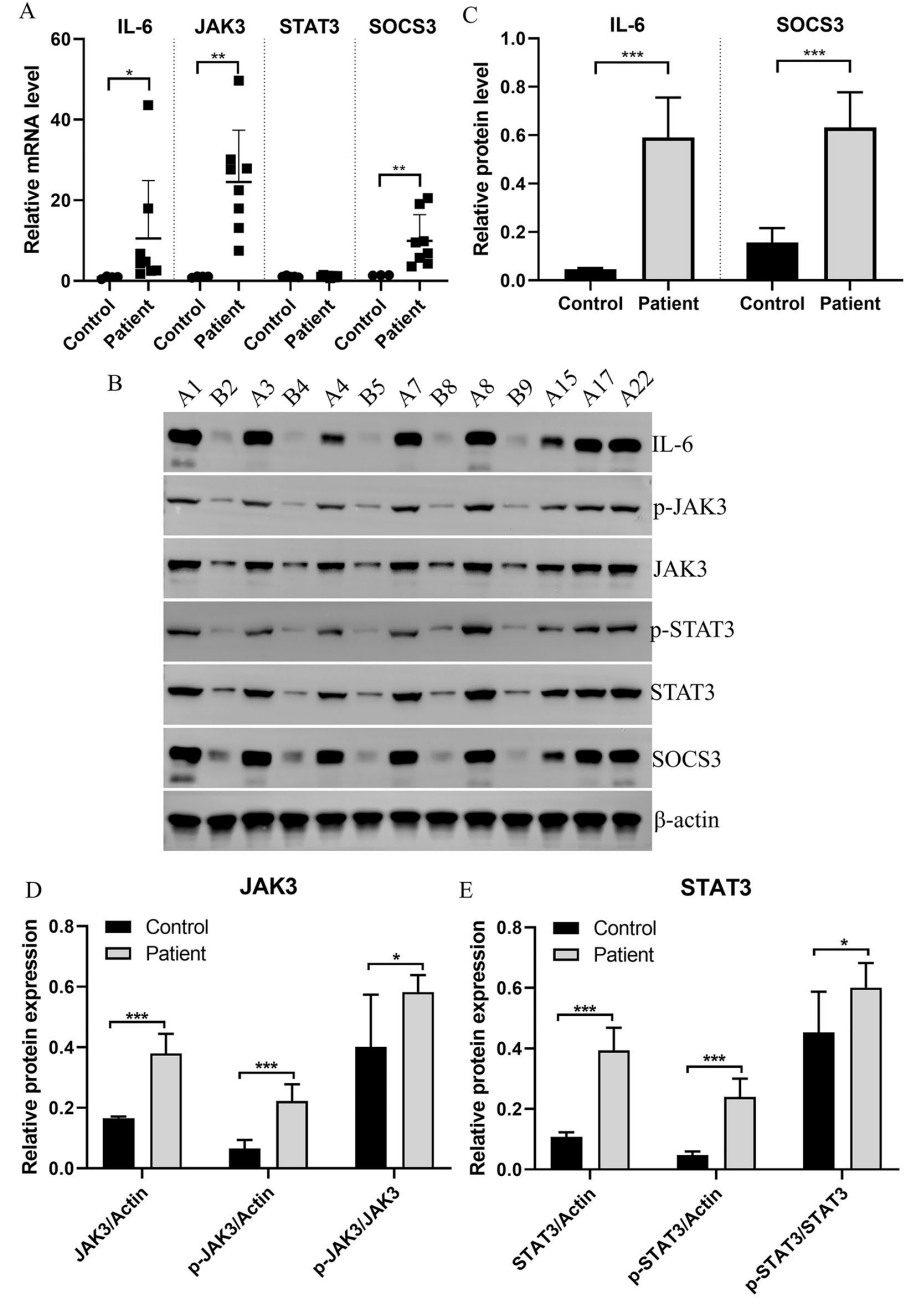
Expressions of JAK/STAT signaling pathway-related proteins (IL-6, JAK3, STAT3, and SOCS3). (**A**) mRNA expressions of the four proteins. (**B**) Protein expressions of IL-6, JAK3, p-JAK3, p-STAT3, STAT3, and SOCS3. A1, A3, A4, A7, A8, A15, A17, and A22 were samples from patients, while B2, B3, B4, B5, B8, and B9 were samples from healthy controls. (**C**) Quantification of the IL-6 and SOCS3 protein expressions. (**D**) Quantification of the p-JAK3 and JAK3 expressions. (**E**) Quantification of the p-STAT3 and STAT3 expressions. ^*^P < 0.05, ^**^P < 0.01, ^***^P < 0.001.

Since JAK3 was the key mRNA and the JAK/STAT signaling pathway was found to be enriched in the bioinformatic analysis, next we sought to detect the expression of the JAK/STAT signaling pathway-related proteins (IL-6, JAK3, STAT3, and SOCS3). As shown in Fig. 5A, the mRNA levels of IL-6, JAK3, and SOCS3 were significantly upregulated in the patient samples compared to the control samples, while the mRNA levels of STAT3 showed no change. Western blot data revealed that compared to the control samples, the protein levels of IL-6, JAK3, STAT3, and SOCS3, as well as the expressions of p-JAK3 and p-STAT3, were significantly upregulated (Figs. 5B–D). Moreover, the relative levels of p-JAK3/JAK3 and p-STAT3/STAT3 were also significantly upregulated (Figs. 5D-E).

## Discussion

In the present study, we performed transcriptomic analysis of skin lesion samples from sporotrichosis patients and samples from healthy skin controls. A total of 671 lncRNAs, 3281 mRNAs, and 214 miRNAs were identified as differentially expressed between the two groups. The DEGs were mainly enriched in various infectious diseases and immune response-related diseases. ceRNA interaction network analysis identified the key mRNAs as JAK3, SLC4A1, ESR1, and GMAT; key lncRNAs as LOC105374338, LOC105370792, and URS0001BCE66F; and key miRNAs as miR-143-5p, miR-20b-5p, miR-31-5p, and miR-1268a.

Our data revealed that the DEGs were involved in many immune-related signaling pathways, including the TLR signaling pathway, NK cell-mediated cytotoxicity, TCR signaling pathway, and NOD-like receptor signaling pathway. When infected with *Sporothrix*, the host commands its whole immune system, including the innate and adaptive immunity elements, to participate in the fight against the invading pathogen. Polysaccharides (i.e., chitin and peptidoglycan) on the cell wall of the fungi can initiate the host’s immune response to the infection when the skin or mucous membrane is invaded by *Sporothrix*^33^. Pattern recognition receptors (PRRs) play a significant role. PRRs are a class of receptors capable of recognizing specific molecular structures on the surfaces of different cells. PRRs expressed on the surfaces of epithelial cells and dendritic cells can help with the recognition of pathogens. PRRs, including Mannose receptors, Dectin-1, toll-like receptors, and NOD-like receptors, have been reported to be involved in the immune process^34,35^. The innate immunity represents the host's first line of defense against pathogens^36^. Innate immune cells play a crucial role in the recognition and eventual elimination of *Sporothrix* in the host. Since the compromised immunity of the host may cause a low or ineffective response to conventional treatments for *Sporothrix*, a combination of immunomodulatory therapies may be needed to deal with the situation. It is worth mentioning that phagocytosis by macrophages and neutrophils, as well as ROS production, is the main innate host defense against *Sporothrix*^2,37,38^.

Toll-like receptors (TLRs) are a class of PRRs that can recognize pathogens or internal danger signals, trigger downstream signaling pathways, and ultimately lead to responses in inflammation and adaptive immunity^39,40^. TLR-2 and TLR-4 have been reported to be involved in the sporotrichosis-induced immune response^41^. Rossato et al.^42^ reported that TLR-2 deficiency resulted in impaired phagocytosis and the impaired production of cytokines, such as TNF-α, IL-6, and IL-10, during *Sporothrix brasiliensis* infection, and that a lack of TLR-2 also resulted in an increased dissemination of *S. brasiliensis* and a polarized Th-17 response to control infection. Guan^43^ et al. found that melanin inhibits the phagocytosis of *S. globosa*, and guards against macrophage attack by providing protection against oxygen- and nitrogen-derived free radicals while inhibiting the host pro-inflammatory cytokine production (TNF-α and IL-6). They also reported that cells with TLR-2/4 knockdown had a reduced killing efficiency toward *S. globosa*. These data indicate that TLR-2 and TLR-4 play important roles in the host defense against *Sporothrix* infection. Our results also showed that the TLR signaling pathway is crucial in the clearance of *Sporothrix* infection, further validating the previous results.

Previous studies have shown that various CD4^+^ T-cell phenotypes in the T-cell receptor (TCR) pathway play an important role in the host defense against fungal infection. The T cell-mediated immune response to *Candida albicans* is a balance between the response of the Th1, Th17, and Treg subsets^44^. The clearance of mucosal surface infections by *C. albicans* is driven by Th17 responses^45^, and the immune responses to oral and cutaneous *C. albicans* are also predominantly driven by Th1 and Th17 cells^46,47^. Th1 cells were reported to be the main specific effector/memory CD4 T-cells response to *Aspergillus fumigatus* in the peripheral blood of healthy people^48^, while Th17 were linked to the response of the effector/memory subsets to lung-derived *Aspergillus*^49^, which suggests that complex adaptive immune responses are involved in the clearance of fungal pathogens. Our findings suggest that the TCR signaling pathway may also play an important role during the development of sporotrichosis; however, the underlying molecular mechanism remains to be elucidated and needs further study.

Inflammasomes are a class of complexes composed of cytoplasmic proteins that mediate the inflammatory response to pathogen infection^50^. Once activated by inflammatory ligands, the inflammasomes activate caspase-1, which promotes maturation of the IL-1β precursor and IL-18 precursor, thereby ultimately inducing pyroptosis^51^. Previous studies have shown that the NOD-like receptor protein 3 (NLRP3) inflammasome plays a critical role against *S. globosa* infection. Yan et al.^35^ treated the footpads of *S. globosa*-infected mice with Nd:YAG 1064 nm laser irradiation, and concluded that the local NLRP3/caspase-1 pyroptosis pathway was activated and promoted Th1/Th17 cell immunity, and the critical effector of pyroptosis caspase-3 was upregulated after the laser treatment. Gonçalves et al.^52^ found that NLRP3, ASC, or caspase-1 knockout mice were more susceptible to *S. schenckii* infection than wild-type mice, suggesting that NLRP3-triggered-inflammatory responses contributed to host protection against infection. Our study further confirmed that the NLR signaling pathway plays an important role in the host immunity to sporotrichosis, for which the molecules involved remain to be further explored.

Our enrichment analysis found that the JAK/STAT pathway was significantly enriched. Moreover, JAK3 was found to be a hub DEG. These data indicate that the JAK/STAT pathway may be important during the development of sporotrichosis. However, whether the JAK/STAT pathway functions in the development of sporotrichosis has not been reported yet. Therefore, we sought to detect the expressions of JAK/STAT pathway-related proteins in sporotrichosis patients. The results showed that the protein expressions of IL-6, JAK3, STAT3, and SOCS3, as well as phosphorylated JAK3 and STAT3, were significantly upregulated, suggesting that the JAK/STAT signaling pathway was activated during the occurrence and development of sporotrichosis. JAKs, a class of non-receptor tyrosine protein kinases that can be activated by many cytokines, activate downstream target genes through STATs and regulate different molecules in many cellular biological processes^53^. SOCS proteins are key regulators of cytokine signaling induced by activated STATs, negatively regulating cytokine signaling through a feedback loop, and have been reported to play a role in the development of infectious diseases^54^. The knockdown of SOCS3 in dendritic cells during *Candida albicans* infection was reported to enhance Th1 differentiation and the Th17 immune response by activating IL-6/STAT3^55^. Here we found SOCS3 was also upregulated, suggesting that the mechanism of the JAK/STAT pathway in sporotrichosis is complex. As has been demonstrated, the JAK-STAT pathway is a double-edged sword, whereby the proper degree of activation helps the host get rid of invading pathogens, while further progression of the disease may occur due to overactivation of the pathway^56^. JAK inhibitors can achieve immunosuppression, which is obtained by decreasing the serum pro-inflammatory factor levels, It should be noted that JAKs are also used to treat rheumatoid arthritis, inflammatory bowel disease, tumors, diabetes, and skin-related diseases^57^. All this evidence suggests that the JAK/STAT pathway may be a therapeutic target for sporotrichosis.

In conclusion, 671 lncRNA DEGs, 3281 mRNA DEGs, and 214 miRNA DEGs were identified to be involved in *S. globosa* infection through the whole transcriptome analysis of sporotrichosis lesion skin tissues. Enrichment analysis showed that these DEGs were mainly involved in the toll-like receptor signaling pathway, T-cell receptor signaling pathway, NOD-like receptor signaling pathway, and JAK/STAT signaling pathway. Further studies are needed to uncover the underlying molecular mechanisms that take place during the occurrence and development of sporotrichosis.

### Supplementary Information

Supplementary Tables.

Supplementary Figures.

Supplementary Information.

## Data Availability

The datasets generated and/or analyzed during the current study are available from the Genome Sequence Archive in National Genomics Data Center, China National Center for Bioinformation / Beijing Institute of Genomics, Chinese Academy of Sciences repository, (https://ngdc.cncb.ac.cn/gsa-human and GSA-Human: HRA003986).
