# Supplementary material for: Whole transcriptome sequencing for revealing the pathogenesis of sporotrichosis caused by Sporothrix globosa
Source: Sci Rep. 2024 Jan 3;14:359. doi: 10.1038/s41598-023-50728-7 (PMC10764346; doi:10.1038/s41598-023-50728-7)
Supplement: Supplementary file 1 — Supplementary Tables. [file 41598_2023_50728_MOESM1_ESM.docx]

**Supplementary Table 1. Primers used in this study**

| **Genes** | **Primers (5’-3’)** |
| --- | --- |
| H-GAPDH-F | TGTTGCCATCAATGACCCCTT |
| H-GAPDH-R | CTCCACGACGTACTCAGCG |
| H- ACTG2-F1 | TCCAAGGATCCCCTCGAGAC |
| H- ACTG2-R1 | AAAGAGCCCCACACAGAGAAG |
| H- SLC7A11-F2 | AATTTCAGGGGCATCGTGGG |
| H- SLC7A11-R2 | TGCTTGAGTTGAGGACCAGT |
| H- JAK3-F1 | GCGAGGCACACGTCAAGAT |
| H- JAK3-R1 | GATTCGGGGGCATACCAGAAA |
| H- DKK1-F4 | TGTGCCTCAGGATTGTGTTGT |
| H- DKK1-R4 | TCTCCACAGTAACAACGCTGG |
| H-IL6-F2 | TGGGCACAGAACTTATGTTGTTC |
| H-IL6-R2 | TGCATAGCCACTTTCCATTATTATT |
| H-stat3-F2 | CTGTGGGAAGAATCACGCCT |
| H-stat3-R2 | ACATCCTGAAGGTGCTGCTC |
| H- SOCS3-F1 | CGGGACAGTCACCGAAAACA |
| H- SOCS3-R1 | AAGATGTCCCGTCTCCTCCA |

**Supplementary Table 2. Demographic characteristics of the study participants**

| Patients | Gender | Age (years) | Duration of disease (month) | Location | Clinical typing |
| --- | --- | --- | --- | --- | --- |
| A1 | M | 77 | 5 | Upper limb | LC |
| A2 | F | 69 | 2 | Face | LC |
| A3 | M | 58 | 12 | Hand | LC |
| A5 | F | 56 | 3 | Face | LC |
| A4 | M | 56 | 4 | Upper limb | FC |
| A6 | F | 53 | 3 | Face | LC |
| A7 | F | 70 | 12 | Upper limb | FC |
| A8 | F | 46 | 3 | Face | FC |
| A10 | M | 77 | 1 | Upper limb | LC |
| A11 | M | 77 | 5 | Upper limb | LC |
| A12 | F | 82 | 3 | Upper limb | LC |
| A13 | F | 65 | 12 | Face | LC |
| A15 | F | 69 | 4 | Face | LC |
| A17 | F | 59 | 4 | Face | FC |
| A22 | F | 79 | 3 | Upper limb | FC |

Note: FC, fixed cutaneous; LC, lymphocutaneous.

**Supplementary Table 3. The top 10 upregulated and downregulated lncRNAs**

| Gene symbol | log2 (samlncRNA / conlncRNA) | Q value |
| --- | --- | --- |
| 'LINC01798' | -8.28038 | 1.39E-09 |
| 'URS0001BD0D87' | -8.07332 | 2.23E-04 |
| 'ISL1-DT' | -7.98746 | 6.02E-19 |
| 'LINC00867' | -7.66993 | 1.65E-08 |
| 'URS0000D5B76D' | -7.39978 | 4.91E-08 |
| 'URS0001BF5131' | -6.79719 | 1.30E-04 |
| 'LOC101926908' | -6.45589 | 6.98E-04 |
| 'URS0001BF80BA' | -6.40244 | 5.02E-05 |
| 'URS0001BE77E4' | -6.37721 | 0.036085 |
| 'LINC01028' | -6.36759 | 0.005648 |
| 'URS00008B9CA5' | 7.591044 | 0.00651 |
| 'URS00008B3675' | 7.601682 | 1.68E-04 |
| 'URS0001BE5D58' | 8.064067 | 4.75E-07 |
| 'URS00009C5820' | 8.190518 | 4.85E-04 |
| 'LINC02154' | 8.206231 | 1.34E-06 |
| 'URS00008B62A7' | 8.428712 | 4.45E-08 |
| 'URS00009C1ADD' | 8.785865 | 1.99E-05 |
| 'URS00008C23F8' | 9.003537 | 2.71E-07 |
| 'URS0001BDD714' | 9.612086 | 2.06E-13 |
| 'URS0000EB1A28' | 9.73502 | 1.34E-17 |

**Supplementary Table 4. The top 10 upregulated and downregulated mRNAs**

| Gene symbol | log2 (samRNA / conmRNA) | Q value |
| --- | --- | --- |
| 'UTP14C' | -22.9853 | 1.42E-09 |
| 'SERTM1' | -10.4979 | 3.42E-23 |
| 'KRT13' | -9.37496 | 1.61E-12 |
| 'CYP1A1' | -8.57712 | 1.17E-06 |
| 'TBX4' | -8.08893 | 1.58E-19 |
| 'EDN3' | -7.87704 | 8.08E-06 |
| 'DLK1' | -7.69802 | 0.019335 |
| 'ISL1' | -7.69795 | 1.37E-16 |
| 'ASB5' | -7.64309 | 1.84E-09 |
| 'ACTC1' | -7.62277 | 2.25E-07 |
| 'FCRL5' | 10.17329 | 3.11E-20 |
| 'LOC102723407' | 10.27289 | 2.59E-14 |
| 'IL24' | 10.56731 | 1.68E-10 |
| 'CXCL8' | 11.17431 | 2.60E-13 |
| 'MMP3' | 11.34981 | 3.43E-31 |
| 'CHIT1' | 11.36888 | 1.28E-15 |
| 'MMP1' | 13.75052 | 8.84E-29 |
| 'SCGB2A2' | 19.62748 | 7.11E-07 |
| 'KRT25' | 21.77215 | 2.57E-08 |
| 'LOC107983983' | 22.24086 | 1.20E-08 |

**Supplementary Table 5. The top 10 upregulated and downregulated miRNAs**

| Gene symbol | log2 (sample5 / control3) | Q value |
| --- | --- | --- |
| 'hsa-miR-133a-3p' | -5.53018 | 6.31E-07 |
| 'hsa-miR-204-3p' | -5.28811 | 6.40E-06 |
| 'hsa-miR-6720-5p' | -5.28525 | 6.59E-04 |
| 'hsa-miR-9983-3p' | -5.14809 | 0.015358 |
| 'hsa-miR-1-3p' | -5.10658 | 4.89E-06 |
| 'hsa-miR-124-3p' | -4.9186 | 0.006352 |
| 'hsa-miR-204-5p' | -4.91717 | 2.88E-05 |
| 'hsa-miR-143-5p' | -4.42528 | 0.001308 |
| 'hsa-miR-6507-5p' | -4.3766 | 0.004343 |
| 'hsa-miR-509-3p' | -4.33009 | 0.003316 |
| 'hsa-miR-549a-5p' | 5.663788 | 5.64E-07 |
| 'hsa-miR-498-5p' | 5.66666 | 0.030498 |
| 'hsa-miR-520f-3p' | 5.898058 | 0.002689 |
| 'hsa-miR-517c-3p' | 5.963258 | 0.001687 |
| 'hsa-miR-520g-3p' | 5.98155 | 0.002215 |
| 'hsa-miR-1283' | 6.114315 | 7.01E-04 |
| 'hsa-miR-549a-3p' | 6.278815 | 9.27E-04 |
| 'hsa-miR-155-3p' | 6.364318 | 3.63E-04 |
| 'hsa-miR-147b-3p' | 6.391381 | 4.37E-04 |
| 'hsa-miR-7702' | 6.993051 | 2.58E-04 |
