# Supplementary material for: Whole transcriptome sequencing for revealing the pathogenesis of sporotrichosis caused by Sporothrix globosa
Source: Sci Rep. 2024 Jan 3;14:359. doi: 10.1038/s41598-023-50728-7 (PMC10764346; doi:10.1038/s41598-023-50728-7)
Supplement: Supplementary file 2 — Supplementary Figures. [file 41598_2023_50728_MOESM2_ESM.docx]

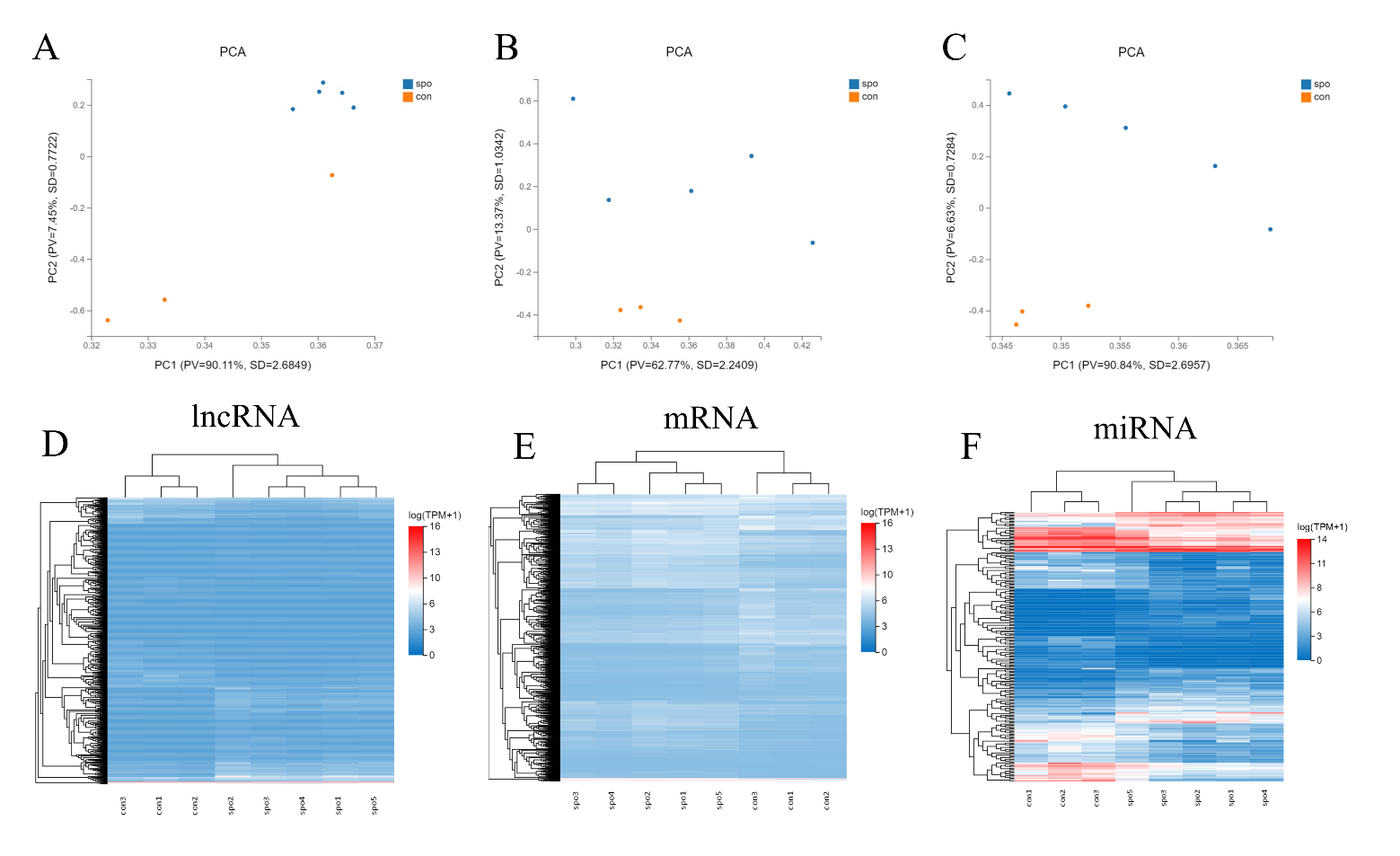


**Supplementary Figure 1. PCA plot (A-C) and heatmap plot (D-E) of the lncRNA (A, D), mRNA (B, E) and miRNA (C, F) expression profiles.**


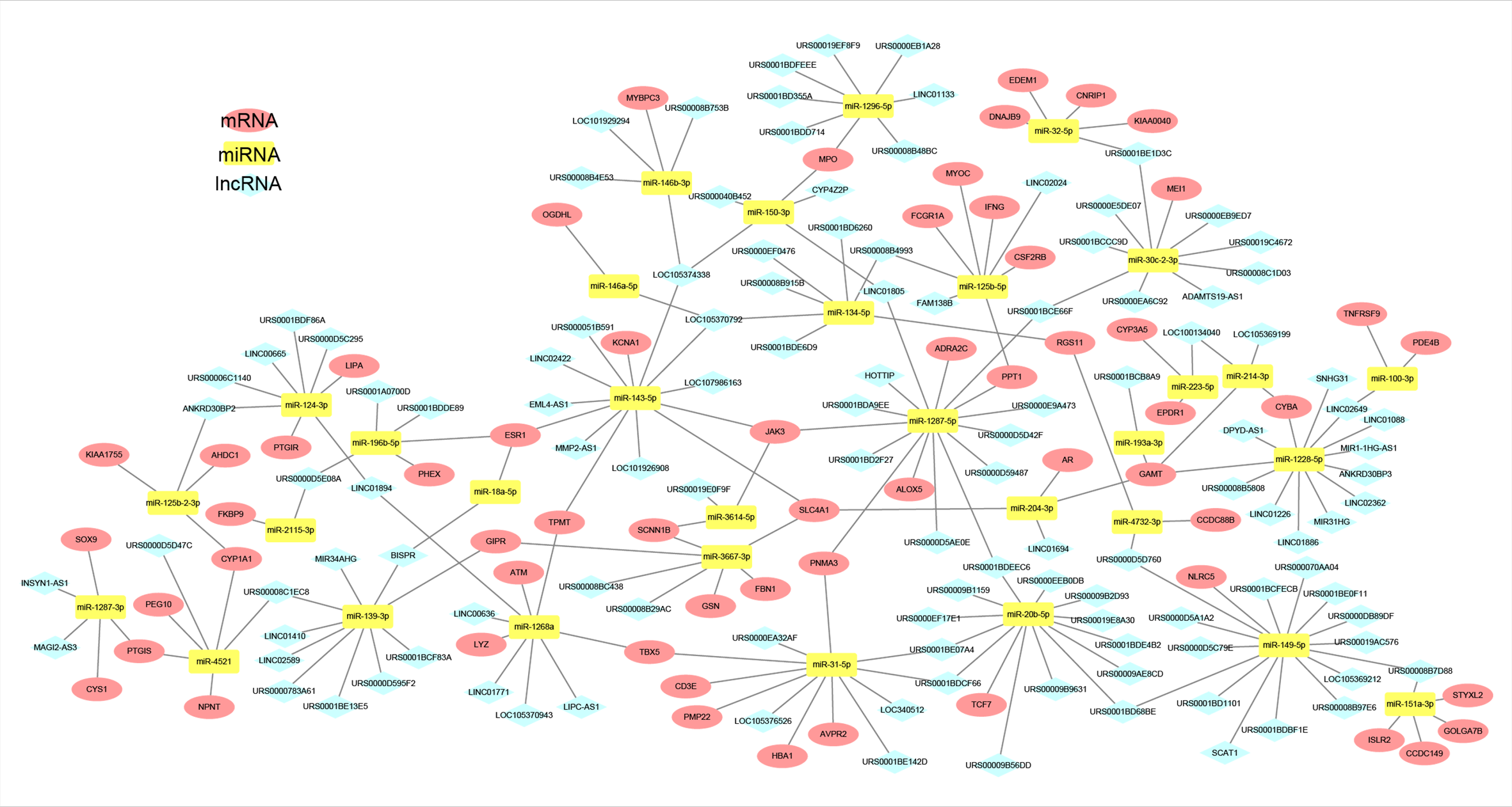


**Supplementary Figure 2. ceRNA interaction network.**


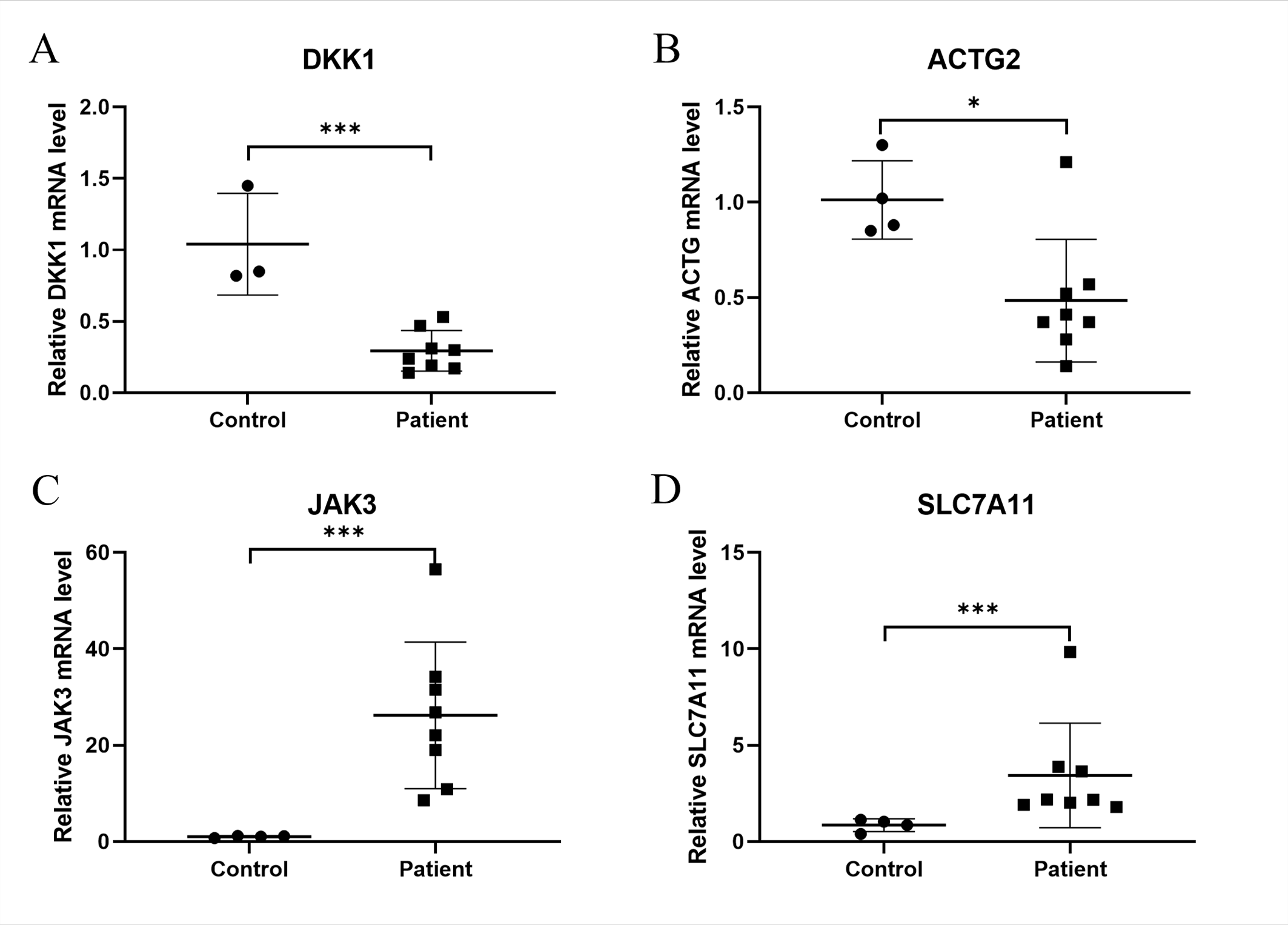


**Supplementary Figure 3. mRNA expression of four DEGs mRNAs.** (A) DKK1; (B) ACTG2; (C) JAK3; (D) SLC7A11. ^*^*P*<0.05, ^***^*P*<0.001.
