# Supplementary figures and images for: Whole transcriptome sequencing for revealing the pathogenesis of sporotrichosis caused by Sporothrix globosa
Source: Sci Rep. 2024 Jan 3;14:359. doi: 10.1038/s41598-023-50728-7 (PMC10764346; doi:10.1038/s41598-023-50728-7)

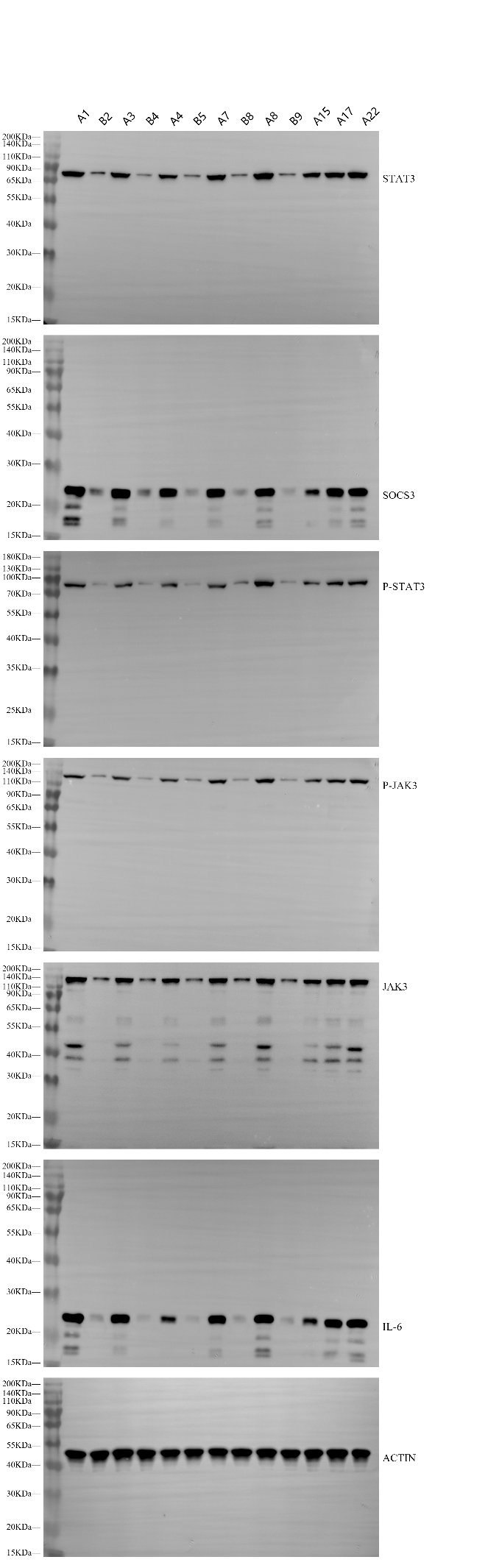

Supplement: Supplementary file 3 — Supplementary Information. [file 41598_2023_50728_MOESM3_ESM.docx]
